# Origin of the *Diplaziumhachijoense* complex (Athyriaceae)

**DOI:** 10.3897/phytokeys.124.35242

**Published:** 2019-06-18

**Authors:** Kiyotaka Hori, Noriaki Murakami

**Affiliations:** 1 The Kochi Prefectural Makino Botanical Garden 4200-6 Godaisan, Kochi 781-8125, Japan The Kochi Prefectural Makino Botanical Garden Kochi Japan; 2 Makino Herbarium, Tokyo Metropolitan University, 1-1 Minami-osawa, Hachioji, Tokyo 192-0397, Japan Tokyo Metropolitan University Hachioji Japan

**Keywords:** apogamous, Athyriaceae, Ferns, *
Diplazium
*, hybridization, phylogeny

## Abstract

We analyzed the phylogeny of the *Diplaziumhachijoense* complex using plastid *trnL-F* and low-copy nuclear marker *AK1* DNA sequences. Based on allele constitution, triploid apogamous species of the *D.hachijoense* complex appeared to have originated from the hybridization of triploid apogamous species and diploid sexual species by recurrent hybridization events. These results suggested that triploid apogamous ferns can achieve hybridization with diploid sexual species by producing diploid spores with irregular meiosis in sporogenesis. Furthermore, the present study predicted the involvement of several unknown species associated with hybridization. More sampling of *Callipteris* species from China and adjacent areas is required to determine the relationships among unknown species and the *D.hachijoense* complex.

## Introduction

In sexually reproductive fern species, meiosis produces 64 haploid spores per sporangium following mitotic divisions of spore mother cells four times, and each mother cell contains half of the parental chromosome number (Manton 1950). By contrast, apogamous ferns produce 32 spores per sporangium by one of two pathways to yield chromosomally unreduced diplospores (Grusz 2016): premeiotic endomitosis (Döpp 1939, Manton 1950) or meiotic first division restitution (Braithwaite 1964). Most apogamous ferns produce their spores by the former pathway (Manton 1950). In addition, a few apogamous species possess a third mechanism of reproduction that yields either 16 or 64 relatively regular shaped spores per sporangium (Park and Kato 2003).

**Figure 1.**
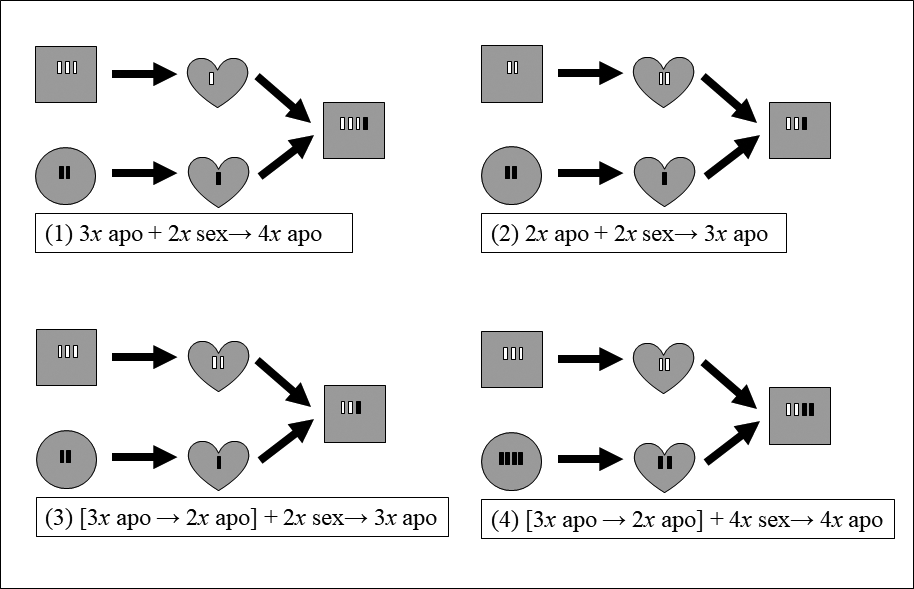
Hybridization patterns with germination from which apogamous species are derived. (**1**) Tetraploid hybrid between a triploid apogamous species and a diploid sexual species, (**2**) triploid hybrid between a diploid apogamous species and a diploid sexual species, (**3**) triploid hybrid between a triploid apogamous species and a diploid sexual species, (**4**) tetraploid hybrid between a triploid apogamous species and a tetraploid sexual species. Circle, sporophyte of sexual species; heart, gametophyte; square, sporophyte of apogamous species.

Apogamous reproduction is not an unusual feature in ferns. Approximately 3% of all fern species (Liu et al. 2012) and 13% of Japanese fern species, for which information regarding their reproductive modes is available, reportedly exhibit apogamous reproduction (Takamiya 1996). Although several apogamous fern species do not require sexual reproduction throughout their life cycles, they exhibit extensive morphological and genetic variation and often form species complexes with continuous morphological variation. Numerous studies have reported reticulate relationships between apogamous and sexual fern species (e.g., Watano and Iwatsuki 1988, Suzuki and Iwatsuki 1990, Lin et al. 1995, Grusz et al. 2009, Chao et al. 2012, Dyer et al. 2012, Ebihara et al. 2012, Hori et al. 2014), which are exhibited in four patterns (Figure 1): (1) tetraploid hybrids are formed between triploid apogamous species and diploid sexual species, (2) triploid hybrids are formed between diploid apogamous species and diploid sexual species, (3) triploid hybrids are formed between triploid apogamous species and diploid sexual species , and (4) tetraploid hybrids are formed between triploid apogamous species and tetraploid sexual species. In patterns (1) and (2), diploid or triploid apogamous species generate unreduced sperm (Walker 1962, Watano and Iwatsuki 1988, Grusz et al. 2009, Chao et al. 2012, Jaruwattanaphan et al. 2013, Dyer et al. 2012) or eggs (Dyer et al. 2012, Hori et al. 2014, Hori et al. 2018a) that are united with a reduced gamete from a sexual species. Alternatively, in patterns (3) and (4), a reduced gamete from a sexual species is united with a reduced diploid sperm or egg generated by an apogamous species (Ebihara et al. 2012, Hori et al. 2014, Hori et al. 2018b, Hori 2018c).

Lin et al. (1992) proposed the hybridization cycle hypothesis as the mechanism of recurrent hybridization that occurs in triploid apogamous species after the discovery of triploid apogamous *Dryopterispacifica*, which produces diploid spores through irregular meiosis. If prothallia of such diploid spores can produce eggs or sperm, an apogamous hybrid species can repeatedly originate from the hybridization of apogamous and sexual species without an increase in ploidy. This hypothesis is supported by numerous reports of irregular meiosis in spore mother cells of triploid Polypodiales apogamous species: *Athyrium* (Athyriaceae, Kurita 1964; Hirabayashi 1970, Park and Kato 2003), *Deparia* (Athyriaceae, Hirabayashi 1970), *Diplazium* (Athyriaceae, Takamiya et al. 1999), *Cyrtomium* (Dryopteridaceae, Hirabayashi 1970), and *Dryopteris* (Dryopteridaceae, Hirabayashi 1967, Lin et al. 1992).

*Diplaziumhachijoense* Nakai (Athyriaceae) is one of the most common triploid apogamous ferns in Japan (Takamiya et al. 1999, Ebihara 2017). It is difficult to identify because of the continuous morphological variation between other closely related triploid apogamous species (*D.conterminum*, *D.dilatatum*, *D.doederleinii*, *D.okinawaense*, *D.taiwanense*, *D.takii*, and *D.virescens*), diploid sexual species (*D.amamianum*), and tetraploid sexual species (*D.nipponicum*). In addition, *D.dilatatum* has a sexual diploid cytotype (Takamiya et al. 1999), and *D.doederleinii* has an apogamous tetraploid cytotype (Takamiya et al. 2001). The ploidy level and reproductive mode of the *D.hachijoense* complex in previous studies are both shown in Table 1. The present study aimed to determine the hybridization patterns between apogamous and sexual species within the *D.hachijoense* complex.

**Table 1.** The ploidy level and reproductive mode of the *D.hachijoense* complex in previous studies.

Species	Reproducive mode	ploidy level	Refereces
* D. conterminum *	apogamous	3×	Takamiya et al. 1999
* D. dilatatum *	apogamous	3×	Takamiya et al. 1999
* D. doederleinii *	apogamous	3×	Takamiya et al. 1999
* D. doederleinii *	apogamous	4×	Takamiya et al. 2001
* D. okinawaense *	apogamous	3×	Takamiya et al. 1999
* D. taiwanense *	apogamous	3×	Takamiya et al. 1999
* D. takii *	apogamous	3×	Takamiya et al. 1999
* D. virescens *	apogamous	3×	Takamiya et al. 1999
* D. amamianum *	sexual	2×	Takamiya et al. 1999
* D. nipponicum *	sexual	4×	Takamiya et al. 1999

## Materials and methods

### Plant materials

In this study, all 10 species of the *Diplaziumhachijoense* complex and an additional four species, which have not yet been assigned scientific names and termed as *Diplazium* sp. 1–4, were investigated. *Diplaziumchinense*, *D.esculentum*, *D.fauriei*, *D.mettenianum*, *Depariajaponica*, *De.viridifrons*, *De.unifurcata*, *Athyriumcrenulatoserrulatum*, and *A.decurrentialatum* were used as outgroups. Voucher information for all samples is listed in Appendix 1. All voucher specimens have been deposited in the Makino Herbarium of Tokyo Metropolitan University, Aichi University of Education, and/or the Kochi Prefectural Makino Botanical Garden.

### Chromosome count and assessment of reproductive modes

We counted mitotic chromosomes from *D.amamianum*, *D.dilatatum*, *D.hachijoense*, *D.nipponicum*, *D.takii*, and *Diplazium* sp. 2–4 (localities are listed in Appendix 1). Chromosome counting methodologies were performed as outlined in Takamiya et al. (1999). To assess the reproductive modes of each sample or herbarium specimen, the number of spores/sporangium were counted. Specimens were considered sexual if the number of spores/sporangium was 64; they were considered apogamous if the number of spores/sporangium was 32 (Manton 1950).

### DNA extraction

For molecular analyses, total DNA was extracted from silica-dried leaves using cetyltrimethylammonium bromide solution, according to Doyle and Doyle (1990).

### Plastid and nuclear DNA sequencing

*trnL-F* was used as the maternally-inherited plastid DNA marker (F: 5'-ATTTGAACTGGTGACACGAG-3' and FernL 1 Ir1: 5'-GGYAATCCTGAGCAAATC-3'; Taberlet et al. 1991, Li et al. 2009). *AK1* (AK4F: 5'-GATGAAGCCATCAAGAAACCA-3' and AKR2: 5'-ATGGATCCAGCGACCAGTAA-3'; Hori et al. 2018b) was used as a biparentally-inherited nuclear marker for polymerase chain reaction-single-strand conformation polymorphism (PCR-SSCP) analysis, which was used to determine allelic variation in each individual (Ebihara et al. 2012, Jaruwattanaphan et al. 2013).

PCR amplification was performed using PrimeSTAR Max DNA Polymerase (Takara, Kyoto, Japan). PCR entailed an initial denaturation step at 95 °C for 10 min, followed by 35 cycles of denaturation, annealing, and elongation steps at 98 °C for 10 s, 55 °C for 5 s, and 72 °C for 5 s, respectively, using a Model 9700 thermal cycler (Applied Biosystems, Foster City, CA, USA).

Gel electrophoresis of *AK1* PCR products was performed using gels containing 2% glycerol at 15 °C for 16 h at 300 V, followed by silver staining. For sequencing of the bands separated on the SSCP gels, the polyacrylamide gel was dried after silver staining by sandwiching the gel between Kent paper and a cellophane sheet on an acrylic back plate at 55 °C for 3 h. To extract the DNA, a piece of the DNA band was peeled from the dried gel using a cutter knife and incubated in 50 μL of Tris-EDTA buffer (10-mM Tris-HCl and 1-mM EDTA, pH 8.0) at 25 °C overnight. The supernatant solution was used as a template for further PCR amplification with the same primer set employed for initial PCR amplification.

PCR products were purified using ExoSAP-IT (USB, Ohio, USA) or Illustra ExoStar 1-Step (GE Healthcare, Wisconsin, USA) and used as templates for direct sequencing. Reaction mixtures for sequencing were prepared using the BigDye Terminator v.3.1 Cycle Sequencing Kit (Applied Biosystems). The reaction mixtures were analyzed using an ABI 3130 Genetic Analyzer (Applied Biosystems). All plant samples were classified based on their PCR-SSCP banding patterns, and each band was DNA sequenced.

### Molecular analysis

For phylogenetic analyses, the sequences were typified and made non-redundant by removing duplicate sequences. Only one sequence representing each allele for *AK1* and for each haplotype for *trnL-F* were used in the datasets (Appendices 1, 2). The sequences were aligned using MUSCLE (Edgar 2004) and assessed with Bayesian inference (BI) analysis using MrBayes 3.2.6 (Ronquist et al. 2012) and maximum parsimony (MP) analysis using the MEGA X software (Kumar et al. 2018). In the BI analysis, the best-fit model of sequence evolution for each DNA region was selected using jModelTest 2.1.10 (Darriba et al. 2012; *trnL-F*: HKY+G model; *AK1*: HKY model). In addition, we assessed BI (*trnL-F*: HKY+I+G model; *AK1*: HKY model) and MP analysis with full-data sets. Four Markov chain Monte Carlo chains were run simultaneously and sampled every 100 generations for 1 million generations in total. Tracer 1.7.1 (Rambaut et al. 2018) was used to examine the posterior distribution of all parameters and their associated statistics, including estimated sample sizes. The first 2,500 sample trees from each run were discarded as burn-in periods. The MP tree was obtained using the subtree pruning-regrafting algorithm (Swofford et al. 1996) at search level 1, at which the initial trees were obtained by the random addition of sequences (10 replicates). Indels were treated as missing characters in the MP and BI analyses. The confidence level of the monophyletic groups was estimated with 1,000 MP bootstrap pseudo-replicates.

## Results

### Chromosome count and estimation of reproductive mode

The ploidy level and reproductive mode of *D.hachijoense* complex species was consistent with previous reports (Takamiya et al. 1999): *D.amamianum* and *D.dilatatum*, 2*n* = 82, diploid sexual; *D.hachijoense* and *D.takii*, 2*n* = 123, triploid apogamous; and *D.nipponicum*, 2*n* = 164, tetraploid sexual (Appendix 1).

### Plastid and nuclear DNA phylogenetic trees

We sequenced 719–748 bp of the *trnL-F* intergenic spacer from different specimens. The aligned *trnL-F* matrix was 748 bp, of which 114 characters (15%) were parsimony-informative. For the *AK1* intron, we sequenced 280–520 bp of the intron for each specimen, yielding a 574 bp aligned matrix, of which 74 characters (13%) were parsimony-informative. The MP trees derived from our *trnL-F* and *AK1* sequence analyses with BI posterior-probabilities (PP) and MP bootstrap percentages (BP) are shown in Figures 2a, 3a, respectively. In the phylogenies with full-data set of *trnL-F* and *AK1* (Figures 2b, 3b, respectively), 118 characters (15%) and 84 characters (14%) were parsimony-informative, respectively.

To define allelic types of the *D.hachijoense* complex, we investigated which diploid sexual or autotriploid apogamous species had each allele supported by PP and BP (Figures 2a–3b and Table 2). Alleles of nuclear genes from samples whose sequences formed a clade with particular lineages were considered to originate from the parental species. Therefore, when two alleles from one triploid apogamous species formed clades with those of parental species A and B, the allele composition of the apogamous species was AB. Unfortunately, PCR-SSCP analysis could not distinguish among the genotypes A1A1B1, A1B1B1, and A1B1 as this method cannot determine the quantity of each allele in PCR products. Therefore, the present study only showed the alleles obtained from each material, not their proportions, in Table 2 and Appendix 1.

To divide each allele number with the alphabet, we used clades supported by BP, PP, and similarity in the sequences. Furthermore, we investigated which diploid sexual or autotriploid apogamous species had each allele (Table 2). Based on allelic relationships shown in Figures 2a–3b and Table 2, *D.dilatatum* seemed to have only type A, *D.takii* had only B, and *D.doederleinii* had only C for both *trnL-F* and *AK1* sequences. Type D containing *D.amamianum* was more clearly monophyletic because the BP and PP values were higher than those for *D.dilatatum*, *D.takii*, and *D.doederleinii*. Regarding other alleles of undetected (or missing) species, we could not conclude which alleles came from the same species. Therefore, we tentatively treated these alleles as individually distinct species, outlining them as E, F, G, H, J and K.

In total, five types of plastid *trnL-F* haplotypes (Type α–ε) and 10 types of nuclear *AK1* alleles (Type A–H, J, and K) were recovered from the *D.hachijoense* complex (Table 2). Plastid haplotypes in the *D.hachijoense* complex were as follows (Fig. 2a, Table 2): sexual or apogamous, type α- *D.dilatatum*, *D.taiwanense*, and *Diplazium* sp. 1; type β- *D.conterminum*, *D.taiwanense*, *D.takii*, and *D.virescens*; type γ- *D.doederleinii* and *Diplazium* sp. 2; type δ- *D.amamianum*, *D.hachijoense*, *D.okinawaense*, *Diplazium* sp. 3, and *Diplazium* sp. 4; and type ε- *D.nipponicum*. Types α, β, γ, and δ were well supported by PP (>0.95) and BP (>90) values. In the phylogeny with full-data set, Type ε was also supported, but Type γ was not supported by PP.

Allelic constitution of *AK1* in the *D.hachijoense* complex were as follows (Fig. 3a, Table 2): sexual or apogamous, type A- *D.dilatatum*; type B- *D.takii*; type C- *D.doederleinii*; type D- *D.amamianum*; *D.taiwanense*, one or two allele A and one allele B; *D.hachijoense*, one allele B and D; *D.okinawaense*, one allele A and B; *D.nipponicum*, one allele D and J; *D.* sp. 1, two allele A and one allele G; *D.* sp. 2, C and K; *D.* sp. 3, C and D; and *D.* sp. 4, D and H. Types A, B, D, and H were rather supported by PP (>0.95) and BP (>70) values. In the phylogeny with full-data set, Type E, J, and K were also supported.

**Figure 2a.**
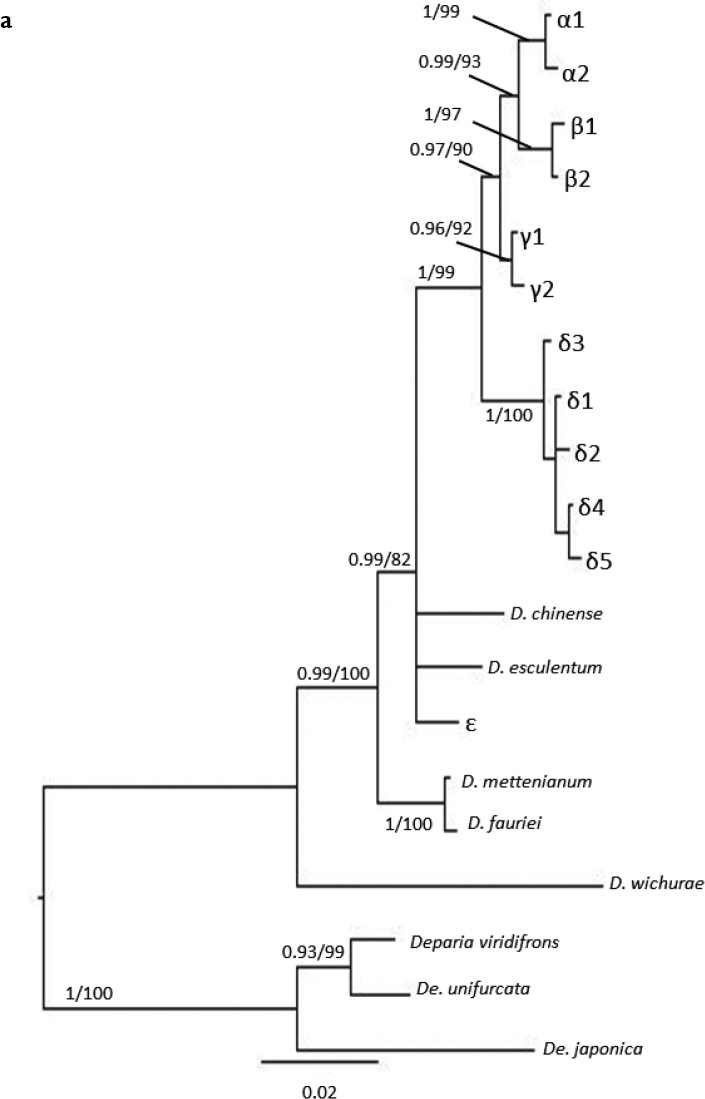
A 50% majority consensus tree resulting from Bayesian Markov chain Monte Carlo Bayesian (B/MCMC) analysis of plastid intergenic spacer *trnL-F* with BIPP (>0.95) and MPBP (>70) node support values. The sequences were typified and made non-redundant by removing duplicate sequences.

**Figure 2b.**
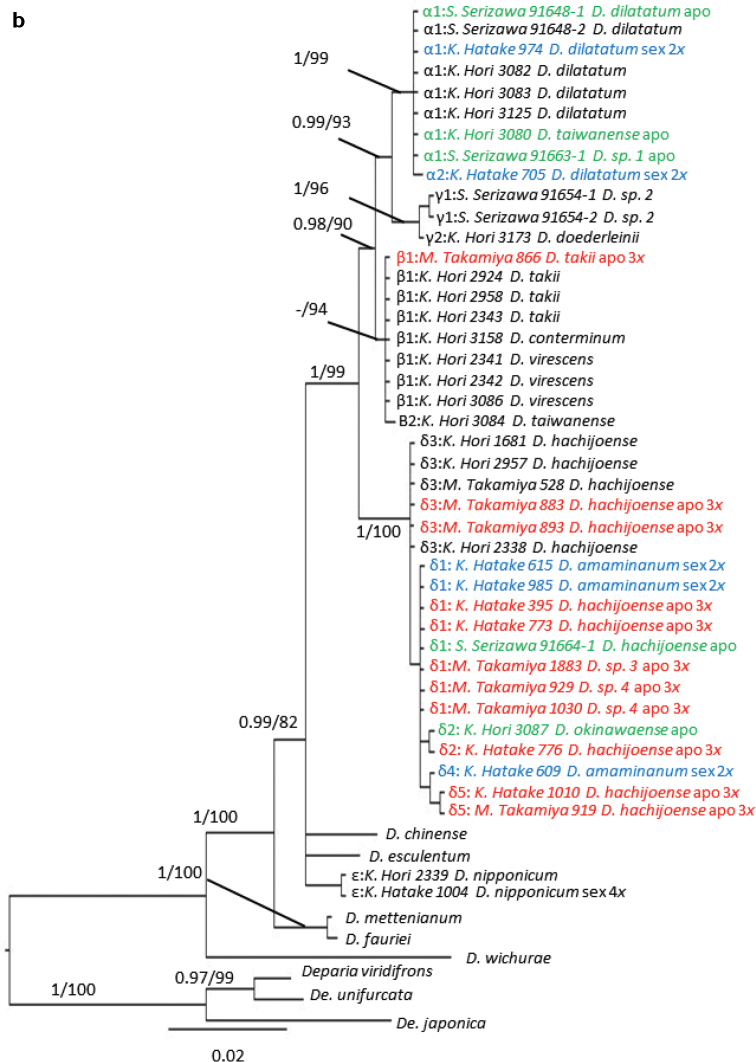
Full-data set of a 50% majority consensus tree resulting from Bayesian Markov chain Monte Carlo Baysean (B/MCMC) analysis of plastid intergenic spacer *trnL-F* with BIPP (>0.95) and MPBP (>70) node support values. Blue, diploid sexual; red, triploid apogamous; green, apogamous but ploidy was not estimated in this study.

**Figure 3a.**
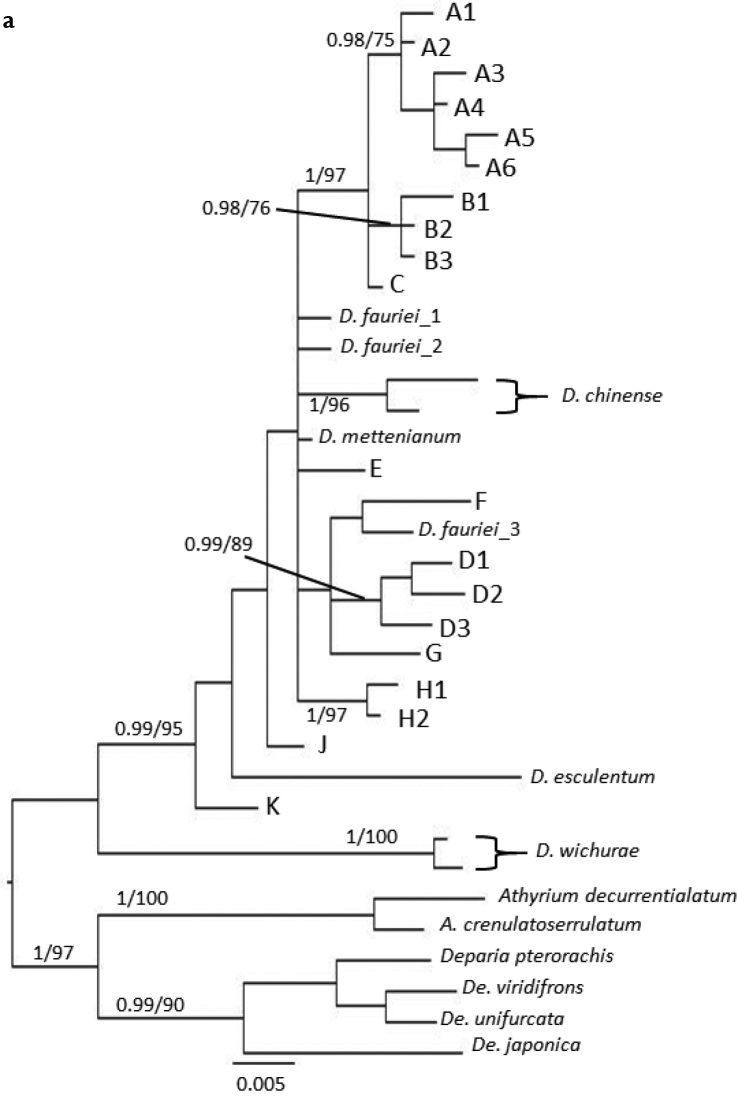
50% majority consensus tree resulting from Bayesian Markov chain Monte Carlo Bayesian (B/MCMC) analysis of the nuclear gene *AK1* with BIPP (>0.95) and MPBP (>70) node support values. The sequences were typified and made non-redundant by removing duplicate sequences.

**Figure 3b.**
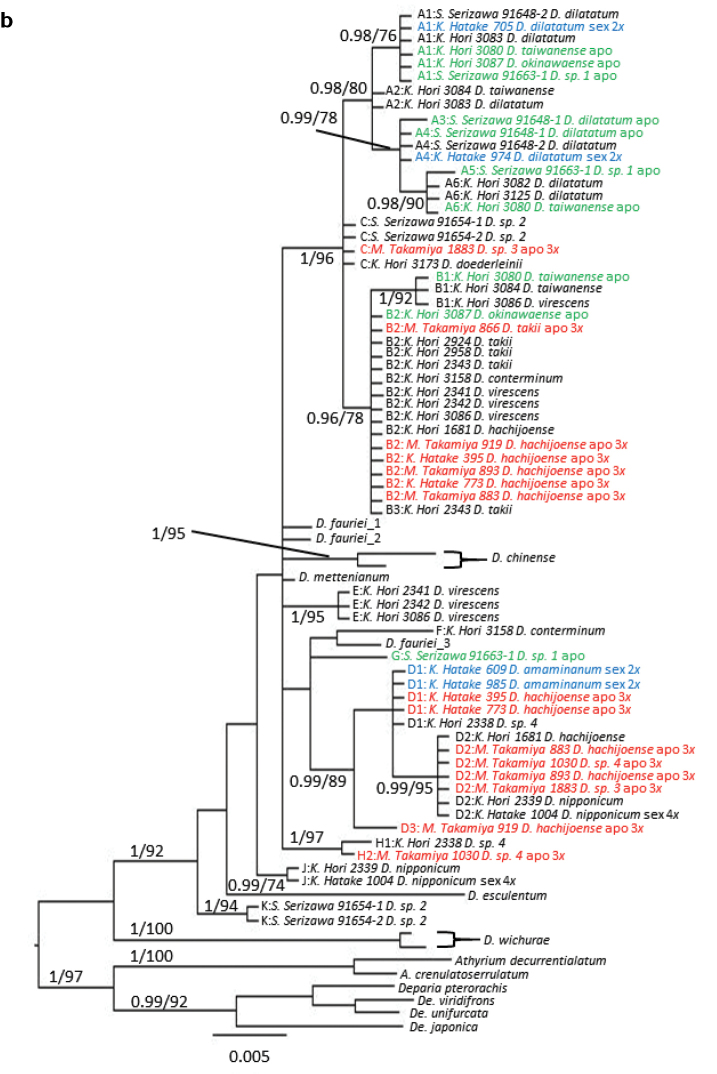
Full-data set of a 50% majority consensus tree resulting from Bayesian Markov chain Monte Carlo Baysean (B/MCMC) analysis of the nuclear gene *AK1* with BIPP (>0.95) and MPBP (>70) node support values. Blue, diploid sexual; red, triploid apogamous; green, apogamous but ploidy was not estimated in this study.

**Table 2.** Reproductive mode, ploidy level, plastid haplpotype (*trnL-F* intergenetic spacer), and nuclear allele (*AK1*) of the *D.hachijoense* complex in this study. Rep, reproductive mode; sex., sexual; apo., apogamous; ploi., ploidy level. Any allelic types of nuclear gene *AK1* that were identified by sequencing are in boldface.

Voucher	Locality	Species	rep.	ploi.	*trnL-F*	*AK1*
*S. Serizawa 91648-1*	Okinawa pref.: Kunigami village, Mt. Yonahadake	* D. dilatatum *	apo.		α1	**A3 and A4**
*S. Serizawa 91648-2*	Okinawa pref.: Nago city, Genka	* D. dilatatum *			α1	**A1A4**
*K. Hatake 705*	Kagoshima pref.: Amami city, Sumiyou village, Santaro-touge, 350m alt.	* D. dilatatum *	sex.	2×	α2	**A1**
*K. Hatake 974*	Kagoshima pref.: Amami city, Naze	* D. dilatatum *	sex.	2×	α1	**A4**
*K. Hori 3082*	Kagoshima pref.: Yakushima Is, Koseda, 70m alt.	* D. dilatatum *			α1	**A6**
*K. Hori 3083*	Kagoshima pref.: Yakushima Is, Koseda, 70m alt.	* D. dilatatum *			α1	**A1A2**
*K. Hori 3125*	Kagoshima pref.: Yakushima Is, Hara, 80m alt.	* D. dilatatum *			α1	**A6**
*M. Takamiya 866*	Mie pref.: Minamimuro county, Kiho-cho	* D. takii *	apo.	3×	β1	**B2**
*K. Hori 2924*	Fukuoka pref.: Kasuya county, Hisayama-machi, 140m alt.	* D. takii *			β1	**B2**
*K. Hori 2958*	Fukuoka pref.: Kasuya county, Hisayama-machi, 140m alt.	* D. takii *			β1	**B2**
*K. Hori 2343*	Mie pref.: Minamimuro county, Kiho-cho, 70m alt.	* D. takii *			β1	**B2B3**
*K. Hori 3173*	Kagoshima pref.: Yakushima Is, Isso-river, 390m alt.	* D. doederleinii *			γ2	**C**
*K. Hatake 615*	Kasgoshima pref.: Amami city, Naze, Honchya-touge, 250m alt.	* D. amamianum *	sex.	2×	δ1	D1
*K. Hatake 985*	Kasgoshima pref.: Amami city, Sumiyou village, Santaro-touge, 350m alt.	* D. amamianum *	sex.	2×	δ1	**D1**
*K. Hatake 609*	Kasgoshima pref.: Amami city, Naze, Ooaza-asato	* D. amamianum *	sex.	2×	δ4	**D1**
*K. Hori 3084*	Kagoshima pref.: Yakushima Is, Koseda, 70m alt.	* D. taiwanense *			β2	**A2B1**
*K. Hori 3080*	Kagoshima pref.: Yakushima Is, Koseda, 71m alt.	* D. taiwanense *	apo.		α1	**A1A6B1**
*K. Hori 3087*	Kagoshima pref.: Yakushima Is, Isso-river, 200m alt.	* D. okinawaense *	apo.		δ2	**A1B2**
*S. Serizawa 91663-1*	Okinawa pref.: Nago city, Genka	*D.* sp. 1	apo.		α1	**A1A5G**
*K. Hori 3158*	Kagoshima pref: Yakushima Is, Tabugawa, 200m alt.	* D. conterminum *			β1	**B2F**
*K. Hori 2341*	Mie pref.: Minamimuro county, Kiho-cho	* D. virescens *			β1	**B2E**
*K. Hori 2342*	Mie pref.: Minamimuro county, Kiho-cho	* D. virescens *			β1	**B2E**
*K. Hori 3086*	Kagoshima pref.: Yakushima Is, Miyanoura river, 20m alt.	* D. virescens *			β1	**B1B2E**
*K. Hatake 773*	Kagoshima pref.: Tokunoshima Is, Mt. Inokawadake, 200m alt.	* D. hachijoense *	apo.	3×	δ1	**B2D1**
*K. Hatake 776*	Kagoshima pref.: Tokunoshima Is, Mt. Inokawadake, 200m alt.	* D. hachijoense *	apo.	3×	δ2	B2D1
*K. Hori 1681*	Chiba pref.: Katori county, Tako-machi, Hayashi	* D. hachijoense *			δ3	**B2D2**
*S. Serizawa 91664-1*	Okinawa pref.: Nago city, Genka	* D. hachijoense *	apo.		δ1	B2D2
*K. Hori 2957*	Fukuoka pref.: Kasuya county, Hisayama-machi, 140m alt.	* D. hachijoense *			δ3	B2D2
*M. Takamiya 528*	Yamaguchi pref.: Nagato city, Ichinoo	* D. hachijoense *			δ3	B2D2
*M. Takamiya 919*	Mie pref.: Minamimuro county, Kiho-cho	* D. hachijoense *	apo.	3×	δ5	**B2D3**
*K. Hatake 1010*	Shizuoka pref.: Shimoda city, Renndaiji-onsenn, 100m alt.	* D. hachijoense *	apo.	3×	δ5	B2D3
*K. Hatake 395*	Kagoshima pref.: Tokunoshima Is, Mt. Inokawadake, 200m alt.	* D. hachijoense *	apo.	3×	δ1	**B2D1**
*M. Takamiya 883*	Mie pref.: Minamimuro county, Kiho-cho	* D. hachijoense *	apo.	3×	δ3	**B2D2**
*M. Takamiya 893*	Mie pref.: Minamimuro county, Kiho-cho	* D. hachijoense *	apo.	3×	δ3	**B2D2**
*M. Takamiya 1883*	Kagoshima pref.: Kagoshima city, Chuzann-cho, Takinoshita-river	*D.* sp. 3	apo.	3×	δ1	**CD2**
*S. Serizawa 91654-1*	Okinawa pref.: Kunigami village, Mt. Yonahadake	*D.* sp. 2			γ1	**CK**
*S. Serizawa 91654-2*	Okinawa pref.: Kunigami village, Mt. Yonahadake	*D.* sp. 2			γ1	CK
*K. Hori 2338*	Mie pref.: Minamimuro county, Kiho-cho	*D.* sp. 4			δ3	**D1H1**
*M. Takamiya 929*	Mie pref.: Minamimuro county, Kiho-cho	*D.* sp. 4	apo.	3×	δ1	D2H2
*K. Hatake 1030*	Kumamoto pref.: Amakusa city	*D.* sp. 4	apo.	3×	δ1	**D2H2**
*K. Hori 2339*	Mie pref.: Minamimuro county, Kiho-cho	* D. nipponicum *			ε	**D2J**
*K. Hatake 1004*	Kanagawa pref.: Minamiashigara city, Kano	* D. nipponicum *	sex.	4×	ε	**D2J**

## Discussion

**Figure 4.**
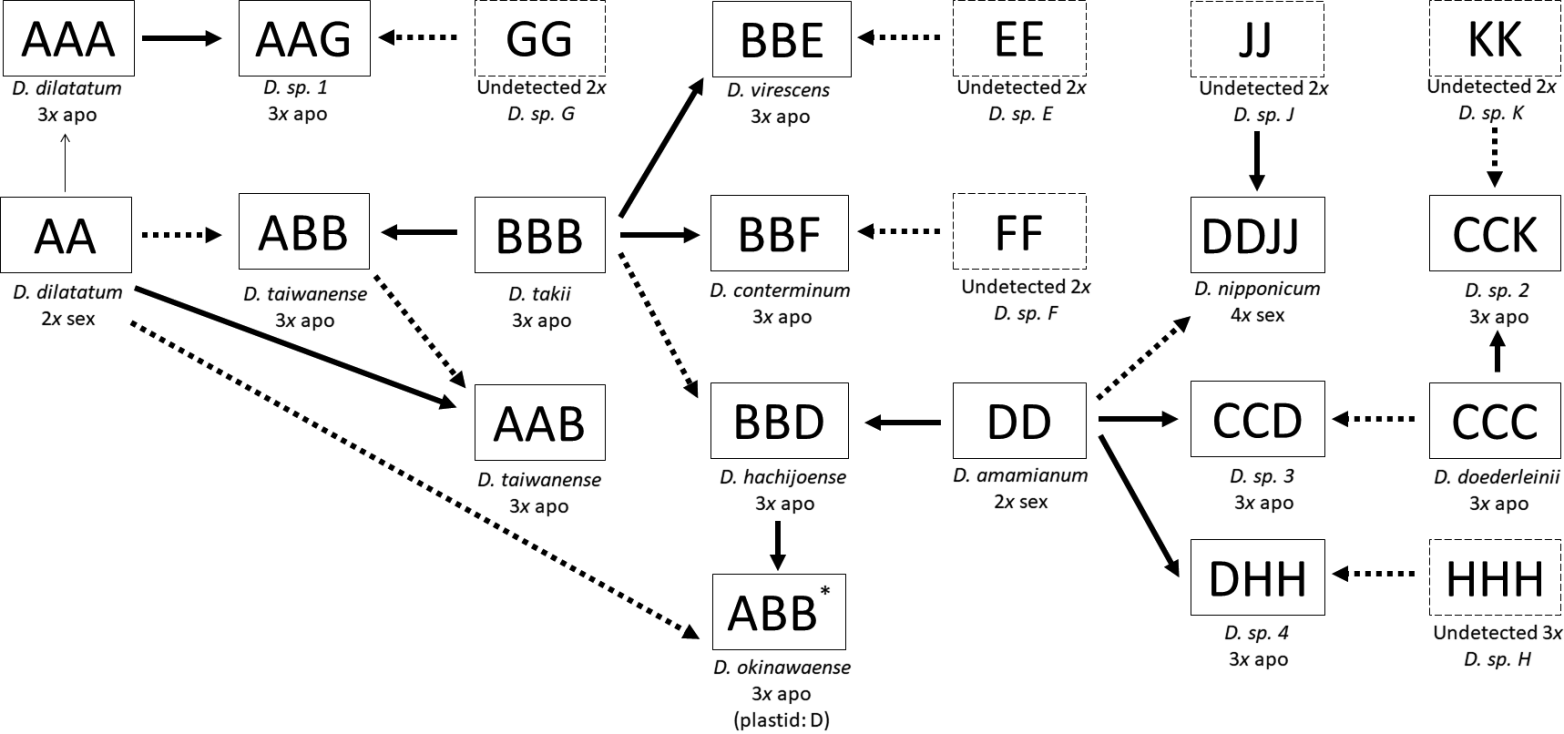
The reticulogram of the *D.hachijoense* complex. Thin-solid arrow, autopolyploidization; Solid arrows, maternal inheritance; dashed arrows, paternal inheritance; square, triploid apogamous or diploid sexual species; dashed square, hypothesized diploid sexual or triploid apogamous species. *D.okinawaense had only nuclear *AK1* allele A of *D.dilatatum* and B of *D.takii*, although plastid haplotype was D of *D.amaminanum*.

Figure 4 represents the reticulogram of the *D.hachijoense* complex. The ploidy levels and reproductive modes of undetected species are unknown; thus, we assigned them as either diploid sexual species (E, F, G, J, and K) or triploid apogamous species (H). If undetected species H was a diploid sexual species, we could not explain the origin of triploid apogamous *Diplazium* sp. 4 (nuclear *AK1*=D and H) because allele D belongs to diploid sexual *D.amamianum*. Thus, it must be a diploid hybrid.

The allelic constitution in *D.hachijoense* suggested that it resulted from hybridization between the diploid sexual species *D.amamianum* and the triploid apogamous species *D.takii*. Because plastid genomes are reported to be maternally-inherited in ferns (Gastony and Yatskievych 1992), *D.amamianum* is likely the maternal parent of *D.hachijoense*. *trnL-F* sequences of *D.amamianum* and *D.hachijoense* are united in Clade δ (Figure 2). The two *AK1* alleles present in *D.hachijoense* are united in Clades B and D, with alleles from *D.takii* and *D.amamianum*, respectively. Furthermore, *D.taiwanense* and *Diplazium* sp. 3 exhibited allelic constitutions to similar to that of *D.hachijoense*, suggesting closely allied progenitors. *Diplaziumtaiwanense* comprises both A and B *AK1* biparentally-inherited alleles. However, one specimen of *D.taiwanense* possessed an α maternally-inherited plastid haplotype derived from *D.dilatatum*, whereas the other specimen of *D.taiwanense* possessed a β plastid haplotype. The former species had one A and B allele, while the latter had two A alleles and one B allele in *AK1*. This suggested recurrent, reciprocal origins of *D.taiwanense*. In the first hybridization event, apogamous *D.takii* is the maternal parent of *D.taiwanense*, whereas sexual *D.dilatatum* is the maternal parent of *D.taiwanense* in the second hybridization event. *Diplazium* sp. 3 has C and D alleles of *AK1* derived from *D.doederleinii* and *D.amamianum*, respectively, and the δ plastid haplotype derived from *D.amamianum*. However, *Diplazium* sp. 3 was difficult to distinguish from *D.hachijoense* because of similar morphological characteristics. Further investigations are required to determine whether *Diplazium* sp. 3 is a novel species.

The allelic constitution of *D.okinawaense* also suggested recurrent hybridization. The *trnL-F* phylogeny suggested that *D.amamianum* is the maternal progenitor of *D.okinawaense*. However, the *AK1* allelic constitution of *D.okinawaense* is A and B, neither of which is found in *D.amamianum*. The inconsistency between the plastid haplotype and the nuclear allelic constitution of *D.okinawaense* may reflect recurrent hybridization events between the triploid apogamous species *D.hachijoense* as the maternal parent and the sexual diploid *D.dilatatum*. Such a scenario may have resulted in the loss of *D.amamianum* nuclear alleles through genetic segregation with recombination (Figure 4).

Allelic constitution in other species suggested that there were six undetected parental species which have only one allele E, F, G, J, K, or H. This study could not resolve ploidy and reproductive mode of these species. Tentatively, in the reticulogram (Figure 4), we proposed *Diplazium* sp. E, F, G, J, and K as hypothesized diploid sexual species and *Diplazium* sp. H as triploid apogamous species. In the reticulogram, we interpreted the origin of six apogamous and one tetraploid sexual species as follows: *D.conterminum* originated from hybridization between apogamous *D.takii* and sexual *Diplazium* sp. E; *D.virescens* originated from *D.takii* and sexual *Diplazium* sp. F; *Diplazium* sp. 1 originated from apogamous *D.dilatatum* and sexual *D.* sp. G; *Diplazium* sp. 2 originated from apogamous *D.doederleinii* and sexual *D.* sp. K; hypothesized diploid sexual *D.* sp. 4 originated from sexual *D.amamianum* and apogamous *D.* sp. H; and tetraploid sexual *D.nipponicum* originated from hybridization between *D.amamianum* and diploid sexual *D.* sp. J. In addition, *Diplazium* sp. 1, *Diplazium* sp. 2, and *Diplazium* sp. 4 were very similar to *D.dilatatum*, *D.doederleinii*, and *D.hachijoense*. More *Callipteris* species (Wei et al. 2013), including members of the *D.hachijoense* complex, need to be collected from China and adjacent areas to further dissect such dynamics.

According to the relationships of diploid sexual species and triploid apogamous species, allelic inheritance patterns in the *D.hachijoense* complex were found to be consistent with the hybridization cycle hypothesis by Lin et al. (1992, 1995) and are similar to examples from other fern taxa. For example, Hori (2018c) reported that *Depariaokuboana* (Athyriaceae) potentially had a hybrid origin from the sexual diploid species *D.viridifrons* and the apogamous triploid species *D.unifurcata*, with the latter producing unreduced diploid sperm. In *Dryopteris* (Dryopteridaceae); several studies have reported that numerous triploid apogamous species share alleles with other triploid apogamous species and with diploid sexual species (Darnaedi et al. 1990, Lin et al. 1992, Hori et al. 2014, Hori et al. 2018b, Hori et al. 2018d). Ebihara et al. (2012) reported that tetraploid apogamous *Dryopterisshibipedis* had alleles in common with apogamous *D.pacifica* and the sexual tetraploid species *D.kinkiensis*.

Morita et al. (1990) reported a similar phenomenon in the angiosperm genus Taraxacum (Asteraceae), revealing that diploid sexual species endemic to Japan hybridize with triploid apogamous species introduced from Europe. The resulting *Taraxacum* are tetraploid or triploid apomicts. Therefore, the hybridization cycle is considered to be an important process that facilitates the production of triploid apogamous hybrids in plant reticulation complexes.

## Conclusions

Continuous morphological variation in the *D.hachijoense* complex reflects a history of recurrent hybridization events among sexual and apomictic taxa, an observation in line with the hybridization cycle hypothesis suggested by Lin et al. (1992, 1995). The resulting hybrid apomict species is comprised of genomes derived from *D.amamianum*, *D.dilatatum*, *D.doederleinii*, and *D.takii*. More analysis of species distributed across China and adjacent areas is required in order to further comprehensively dissect the relationships between unknown lineages, undescribed species, and all members of the *D.hachijoense* complex.

## Appendix 1

**Voucher specimens examined in this study.** Any allelic types of nuclear gene *AK1* that were identified by sequencing are in boldface. Otherwise, the allelic types of nuclear gene *AK1* were deduced from comparisons of band positions in SSCP gels. Data are in the order: Species name – locality, voucher (Herbarium), reproductive mode (sex: sexual, apo: apogamous), chromosome number, ploidy, haplotype of plastid *trnL-F*, allele of nuclear *AK1*. The haplotype of outgroups was only shown in Appendix 2 with accession numbers.

**Members of the *D.hachijoense* complex**

***Diplaziumamamianum* Tagawa** – JAPAN. **Kasgoshima pref.**: Amami city, Naze, Honchya-touge, 250m alt., May 7 2017, *K. Hatake 615* (KUMA), sex, 2*n* = 82, 2*x*, δ1, D1; ibid., Sumiyou village, Santaro-touge, 350m alt., May 26, 2018, *K. Hatake 985* (KUMA), sex, 2*n* = 82, 2*x*, δ1, **D1**; ibid., Naze, Ooaza-asato, May 6, 2017, *K. Hatake 609* (KUMA), sex, 2*n* = 82, 2*x*, δ4, **D1.**

***Diplaziumconterminum* Christ** – JAPAN. **Kagoshima pref**: Yakushima Is, Tabugawa, 200m alt., 130°36'55.92"N, 30°23'19.23"E, January 23 2019, *K. Hori 3158* (MBK), β1, **B2F**.

***Diplaziumdilatatum* Blume** – JAPAN. **Kagoshima pref.**: Amami city, Sumiyou village, Santaro-touge, 350m alt., September 7, 2017, *K. Hatake 705* (KUMA), sex, 2*n* = 82, 2*x*, α2, **A1**; ibid., Naze, May 24, 2018, *K. Hatake 974* (KUMA), sex, 2*n* = 82, 2*x*, α1, **A4**; ibid., Yakushima Is, Koseda, 70m alt., 130°39'24.54"N, 30°22'44.24"E, January 20 2019, *K. Hori 3082* (MBK), α1, **A6**; ibid.; *K. Hori 3083* (MBK), α1, **A1A2**; ibid., Hara, 80m alt., 130°34'19.72"N; 30°14'41.56"E, January 22 2019, *K. Hori 3125* (MBK), α1, **A6**. **Okinawa pref.**: Kunigami village, Mt. Yonahadake, *S. Serizawa 91648-1* (AICH), apo, α1, **A3A4**; Nago city, Genka, *S. Serizawa 91658-2* (AICH), α1, **A1A4.**

***Diplaziumdoederleinii* (Luerss.) Makino** – JAPAN. **Kagoshima pref.**: Yakushima Is, Isso-river, 390m alt., 130°29'22.32"N; 30°25'13.09"E, January 23 2019, *K. Hori3173* (MBK), apo, γ2, **C.**

***Diplaziumhachijoense* Nakai** – JAPAN. **Chiba pref.**: Katori county, Tako-machi, Hayashi, October 25 2014, *K. Hori 1681* (MAK), δ3, **B2D2. Kagoshima pref.**: Tokunoshima Is, Mt. Inokawadake, 200m alt., September 12 2016, *K. Hatake 395* (KUMA), apo, 2*n* = 123, 3*x*, δ1, **B2D1**; ibid., January 13 2018, *K. Hatake 773* (KUMA), apo, 2*n* 123, 3*x*, δ1, **B2D1**; ibid., January 13 2018, *K. Hatake 776* (KUMA), apo, 2*n* = 123, 3*x*, δ2, B2D1. **Mie pref.**: Minamimuro county, Kihou-cho, March 18 2018, *M. Takamiya 883, 893* (KUMA), apo, 2*n* = 123, 3*x*, δ3, **B2D2**; ibid., *M. Takamiya 919* (KUMA), apo, 2*n* = 123, 3*x*, δ5, **B2D3. Okinawa pref.**: Nago city, Genka, *S. Serizawa 91664-1* (AICH), δ1, B2D1. **Shizuoka pref.**: Shimoda city, Renndaiji-onsenn, 100m alt., 138°55'16.85"N; 34°41'55.23"E, May 22 2018, *K. Hatake 1010* (KUMA), apo, 2*n* = 123, 3*x*, δ5, B2D3. **Yamaguchi pref.**: Nagato city, Ichinoo, January 4 1996, *M. Takamiya 528* (KUMA), apo, 2*n* = 123, 3*x*, δ3, B2D2.

***Diplaziumnipponicum* Tagawa** – JAPAN. **Kanagawa pref.**: Minamiashigara city, Kano, March 18 2018, *M. Takamiya 1004* (KUMA), sex, 2*n* = 164, 4*x*, ε, **D2J. Mie pref.**: Minamimuro county, Kiho-cho, 70m alt., 135°59'29.5"N; 33°45'55.2"E, July 6 2016, *K. Hori 2339* (MAK), ε, **D2J.**

***Diplaziumokinawaense* Tagawa** – JAPAN. **Kagoshima pref.**: Yakushima Is, Isso-river, 200m alt., 130°28'21.08"N; 30°26'23.30"E, Jan 21 2019, *K. Hori3087* (MBK), apo, δ2, **A1B2.**

***Diplaziumtaiwanense* Tagawa** – JAPAN. **Kagoshima pref.**: Yakushima Is, Koseda, 70m alt., 130°39'24.54"N; 30°22'44.24"E, January 20 2019, *K. Hori 3080* (MBK), apo, α1, **A1A6B1**; ibid., 70m alt., 130°39'24.54"N; 30°22'44.24"E, January 20 2019, *K. Hori 3084* (MBK), β2, **A2B1**.

***Diplaziumtakii* Sa.Kurata** – JAPAN. **Mie pref.**: Minamimuro county, Kiho-cho, 70m alt., 135°59'29.5"N; 33°45'55.2"E, July 6 2016, *K. Hori 2343* (MAK), β1, **B2B3**; ibid.; *M. Takamiya 866* (KUMA), apo, 2*n* = 123, 3*x*, β1, **B2. Fukuoka pref.**: Kasuya county, Hisayama-machi, 140m alt., 130°32'2.27"N; 33°40'44.18"E, July 6 2016, *K. Hori 2924, 2958* (MBK), β1, **B2.**

***Diplaziumvirescens* Kunze** – JAPAN. **Mie pref.**: Minamimuro county, Kiho-cho, 70m alt., 135°59'29.5"N; 33°45'55.2"E, July 6 2016, *K. Hori 2341, 2342* (MAK), β1, **B2E. Kagoshima pref.**: Yakushima Is, Miyanoura river, 20m alt., 130°32'31.91"N; 30°24'48.36"E, Jan 21 2019, *K. Hori 3086* (MBK), β1, **B1B2E**.

***Diplazium* sp. 1** – JAPAN. **Okinawa pref.**: Nago city, Genka, *S. Serizawa 91663-1* (AICH), apo, α1, **A1A5G.**

***Diplazium* sp. 2** – JAPAN. **Okinawa pref.**: Kunigami village, Mt. Yonahadake, *S. Serizawa 91654-1, S. Serizawa 91654-2* (AICH), γ1, **CK.**

***Diplazium* sp. 3** – JAPAN. **Kagoshima pref.**: Kagoshima city, Chuzann-cho, Takinoshita-river, *M. Takamiya 1883* (KUMA), apo, 2*n* = 123, 3*x*, δ1, **CD2.**

***Diplazium* sp. 4** – JAPAN. **Mie pref.**: Minamimuro county, Kiho-cho, 70m alt., 135°59'29.5"N; 33°45'55.2"E, July 6 2016, *K. Hori 2338* (MAK), δ3, **D1H1**; ibid., March 18 2018, *M. Takamiya 929* (KUMA), apo, 2*n* = 123, 3*x*, δ1, D2H2. **Kumamoto pref.**: Amakusa city, June 14 2018, *K. Hatake 1030* (KUMA), apo, 2*n* = 123, 3*x*, δ1, **D2H2.**

**Outgroups**

***Diplaziumchinense* (Baker) C.Chr.** – JAPAN. **Kochi pref.**: Agawa county, Niyodogawa-cho, Iwayagawa, 250m alt., 133°03'43.15"N; 33°32'22.21"E, June 16 2018, *K. Hori 3023* (MBK).

***Diplaziumesculentum* (Retz.) Sw.** – JAPAN. **Kagoshima pref.**: Isa city, Oguchisogi, *M. Takamiya 1109* (MBK).

***Diplaziumfauriei* Christ** – JAPAN. **Okinawa pref.**: Kunigami village, Mt. Yonahadake, *S. Serizawa 91656-1* (AICH).

***Diplaziummettenianum* (Miq.) C.Chr.** – JAPAN. **Mie pref.**: Minamimuro county, Kiho-cho70m alt., 135°59'29.5"N; 33°45'55.2"E, July 6 2016, *K. Hori 2336* (MAK).

***Diplaziumwichurae* (Mett.) Diels** – JAPAN. **Kanagawa pref.**: Zushi city, Jinnmuji, 60m alt., 139°36'18.19"N, 35°18'14.71"E, July 6 2016, *K. Hori 1763* (MAK).

***Depariajaponica* (Thunb.) M.Kato** – JAPAN. **Kyoto pref.**: Sakyo-ku, Kibune, 300m alt., 135°45'50.79"N; 35°7'30.85"E, July 14 2018, *K. Hori 3031* (MBK).

***Depariaunifurcata* (Baker) M.Kato** – JAPAN. **Kochi pref.**: Agawa county, Niyodogawa-cho, Iwayagawa, 250m alt., 133°03'43.15"N; 33°32'22.21"E, June 16 2018, *K. Hori 3021* (MBK).

***Depariaviridifrons* (Makino) M.Kato** – JAPAN. **Kochi pref.**: Takaoka county, Ochi-cho, May 30 2018, *K. Hori 2971* (MBK).

***Athyriumcrenulato-serrulatum* Makino** – JAPAN. **Tokyo pref.**: Ome city, Mt. Mitake, 880m alt., 139°08'32.09"N; 35°48'12.62"E, June 4 2018, *K. Hori 2979* (MBK).

***Athyriumdecurrenti-alatum* (Hook.) Copel.** – JAPAN. **Tokyo pref.**: Hachioji city, Komagino, June 5 2018, *K. Hori 2986* (MBK).

## Appendix 2

**DNA data accession numbers of the obtained nucleotide sequences used for construction of molecular phylogenetic trees in this study.** Data quoted from Hori (2018c) are marked by asterisks.

***AK1***

A1, LC468160; A2, LC468161; A3, LC468162; A4, LC468163; A5, LC468164; A6, LC468165; B1, LC468167; B2, LC468168; B3, LC468169; C, LC468166; D1, LC468172; D2, LC468173; D3, LC468174; E, LC468170; F, LC468171; G, LC468175; H1, LC468180; H2, LC468181; J, LC468176; K, LC468177; *Diplaziumchinense*, LC468179, LC468182; *D.esculentum*, LC468186; *D.fauriei*, LC468183- LC468185; *D.mettenianum*, LC468178; *D.wichurae*, LC468187, LC468188; *Depariajaponica*, LC431726*; *D.unifurcata*, LC421961*; *D.viridifrons*, LC421960*; *Athyriumcrenulatoserrulatum*, LC421516*; *A.decurrentialatum*, LC421512*.

***trnL-F***

α1, LC468195; α2, LC468196; β1, LC468199; β2, LC468200; γ1, LC468197; γ2, LC468198; δ1, LC468201; δ2, LC468202; δ3, LC468203; δ4, LC468204; δ5, LC468205; ε, LC468208; *Diplaziumchinense*, LC468193; *D.esculentum*, LC468189; *D.fauriei*, LC468206; *D.mettenianum*, LC468207; *D.wichurae*, LC468190; *Depariajaponica*, LC468194; *D.viridifrons*, LC468191; *D.unifurcata*, LC468192.
